# Supplementary material for: Strong intrinsic room-temperature ferromagnetism in freestanding non-van der Waals ultrathin 2D crystals
Source: Nat Commun. 2021 Sep 28;12:5688. doi: 10.1038/s41467-021-26009-0 (PMC8478980; doi:10.1038/s41467-021-26009-0)
Supplement: Supplementary file 1 — Supplementary information. [file 41467_2021_26009_MOESM1_ESM.pdf]

Supplementary Information for

Strong intrinsic room-temperature ferromagnetism in freestanding non-van der Waals ultrathin 2D crystals

Hao Wu[1], Wenfeng Zhang[1], Li Yang[1], Jun Wang[2], Jie Li[1], Luying Li[3], Yihua Gao[3,4], Liang Zhang,[5] Juan Du[6,7], Haibo Shu[2], Haixin Chang[1,5,8]*

[1]Center for Joining and Electronic Packaging, State Key Laboratory of Material Processing and Die & Mold Technology, School of Materials Science and Engineering, Huazhong University of Science and Technology (HUST), Wuhan 430074, China.

[2]College of Optical and Electronic Technology, China Jiliang Univeristy, Hangzhou 310018, China.

[3]Center for Nanoscale Characterization and Devices, Wuhan National Laboratory for Optoelectronics, Huazhong University of Science and Technology, Wuhan 430074, China.

[4]School of Physics, Huazhong University of Science and Technology, Wuhan 430074, China.

[5]School of Science and Center for Materials Science and Engineering, Guangxi University of Science and Technology, Liuzhou, China.

[6]Ningbo Institute of Material Technology & Engineering, Chinese Academy of Sciences, Ningbo 315201, China.

[7]Institute of Materials, Shanghai University, Shanghai 200444, China.

[8]Institute for Quantum Science and Engineering, Huazhong University of Science and Technology, Wuhan 430074, China.

*Corresponding author. Email: hxchang@hust.edu.cn

This Supplementary Information file includes:

Supplementary Figs. 1-23 and Supplementary Table 1.

Supplementary Discussions

Supplementary References

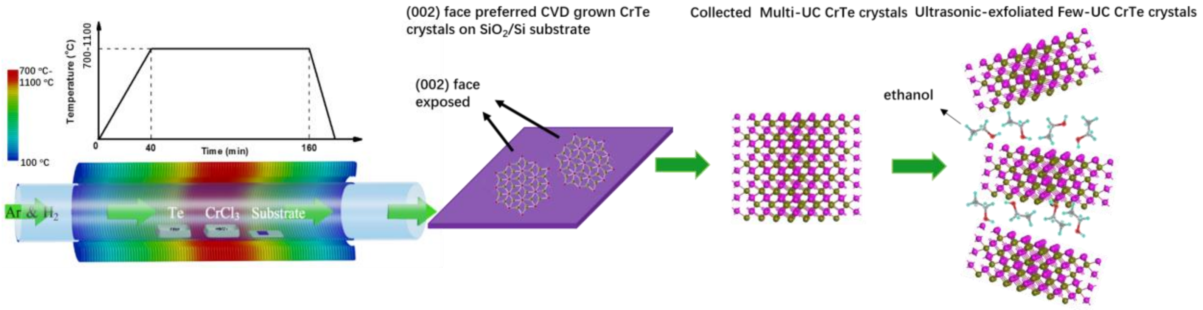

Supplementary Fig. 1. Scheme of the CVD-assisted ultrasonication method for ultrathin 2D CrTe crystals. The purple and brown balls represent Cr and Te atoms, respectively.

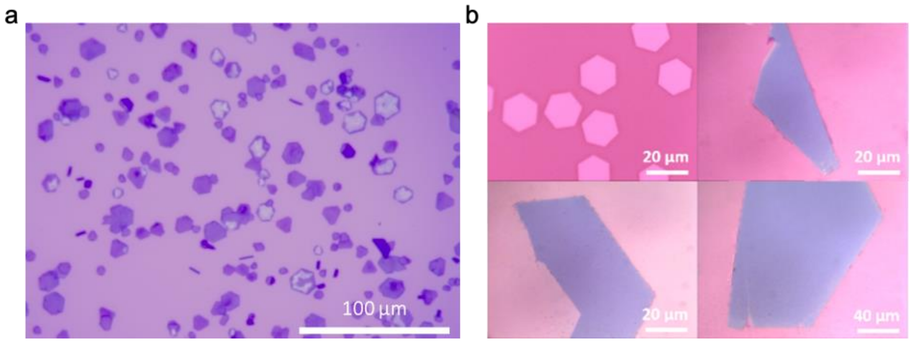

Supplementary Fig. 2. Crystals distribution of CVD grown CrTe crystals on the SiO_2/Si substrate. a, b, Optical images of the general different thickness CrTe crystals area (**a**) and the majority thin crystals areas (**b**) for direct CVD grown CrTe crystals.

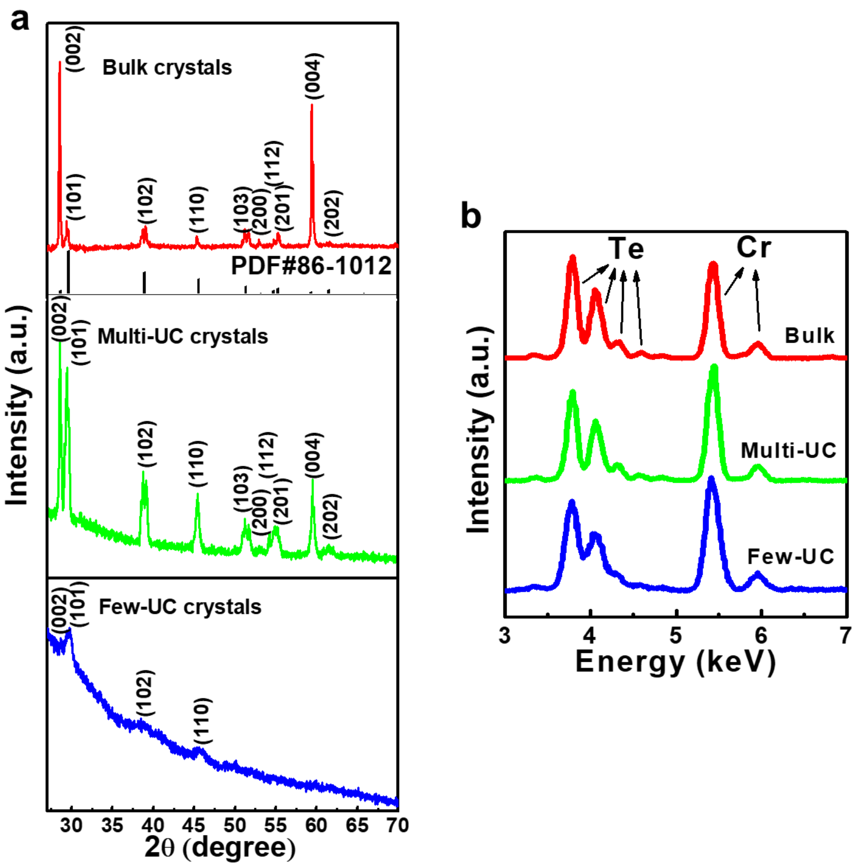

Supplementary Fig. 3. Characterizations of different thickness CrTe crystals. a, X-ray diffraction (XRD) spectra of bulk, multi-UC and few-UC crystals. **b,** XRF spectra of bulk, multi-UC, few-UC CrTe crystals.

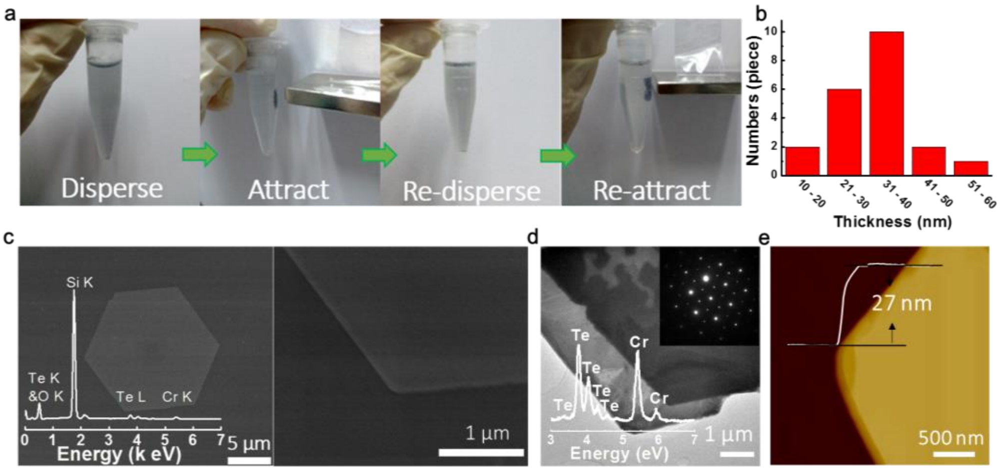

Supplementary Fig. 4. Multi-UC CrTe crystal characterizations. a, The collected multi-UC CrTe crystals homo-dispersed in ethanol are easily attracted by a common magnet and can re-disperse again. **b,** Thickness distribution of multi-UC CrTe crystals from AFM statistical analysis. **c,** SEM images of a multi-UC CrTe single crystal grown on SiO_2/Si substrate (left) and its amplifying edges (right). **d,** TEM image for multi-UC CrTe crystals with EDX spectra and selected-area electron diffractions. **e,** AFM image for a multi-UC CrTe crystal with thickness about 27 nm.

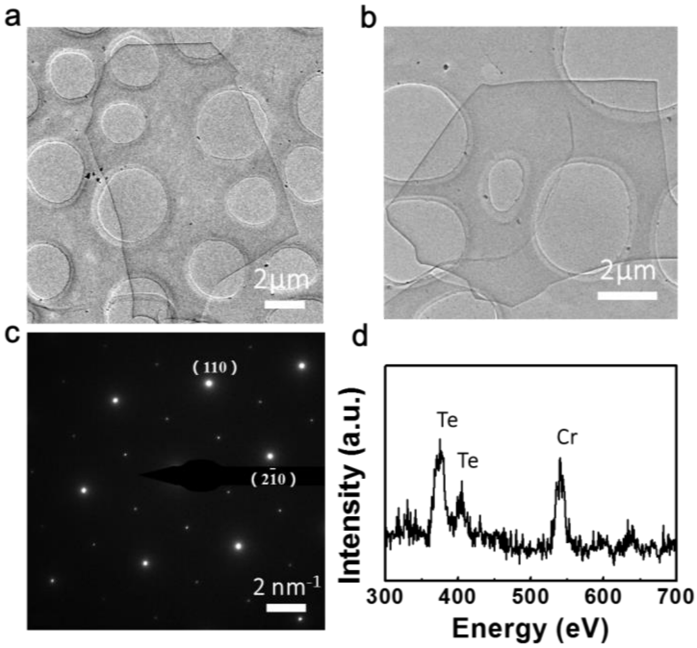

Supplementary Fig. 5. Few-UC CrTe crystal characterizations. a, b, Micrometer-Scale TEM pictures of the few-UC CrTe crystals. **c, d,** The selected-area electron diffraction (SAED) (**c**) and the EDX spectrum (d) on the few-UC CrTe crystal.

Notes:

The atomic ratios of Cr: Te in obtained multi- and few-UC CrTe crystal are similar with bulk crystal and very close to 1: 1 for typical stoichiometry of CrTe (Supplementary Fig. 3b, 5d). Selected-area electron diffraction (SAED) (Fig. 2f, Supplementary Fig. 4d inset, Supplementary Fig. 5c) exhibits two-folds hexagonal diffraction spots, indicating the P63/mmc symmetry superstructure of single crystal 2D CrTe. Element mapping of Cr and Te by energy-dispersive X-ray spectroscopy (EDX) in a few-UC CrTe crystal confirms the atomic homogeneity in the few-UC crystal (Fig. 2d). From the X-ray diffraction (XRD) spectra (Supplementary Fig. 3a), the multi- and few-UC CrTe crystals show XRD patterns that match well with the standard NiAs-type CrTe crystals, similar with the bulk CrTe crystals. The intensities of (002) and (004) faces are higher than others in multi-UC crystals, indicating a (002) face preferred growth. The relatively weaker and broadened peaks in few-UC CrTe crystals result from the ultrathin thickness and smaller lateral size.

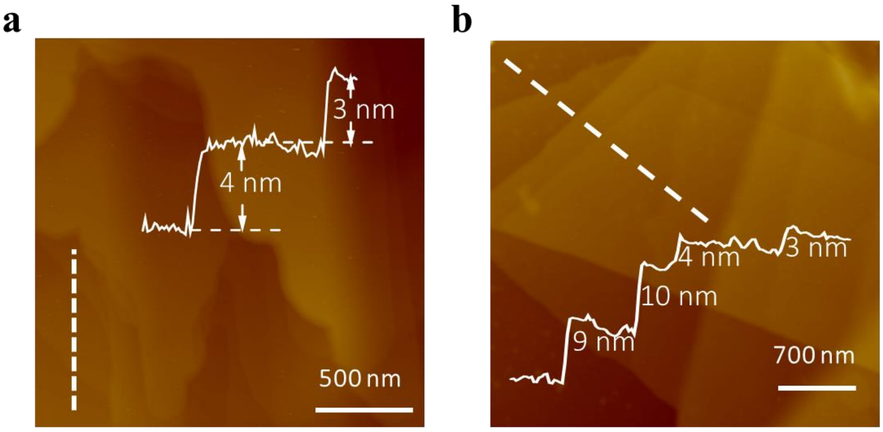

Supplementary Fig. 6. AFM images of stacks of exfoliated CrTe nanosheet crystals, confirming the nanosheet structures.

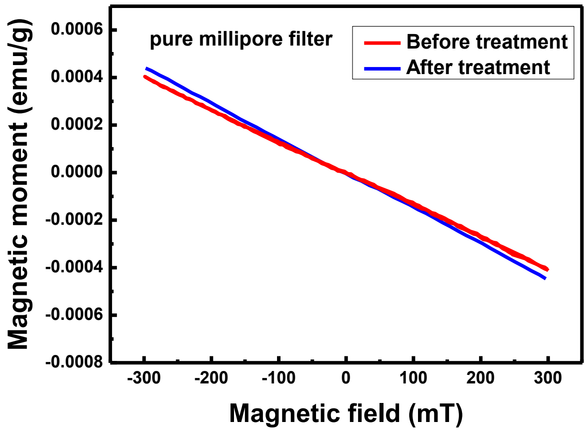

Supplementary Fig. 7. Diamagnetic property of pure millipore filter blank substrate before and after treatment by ethanol.

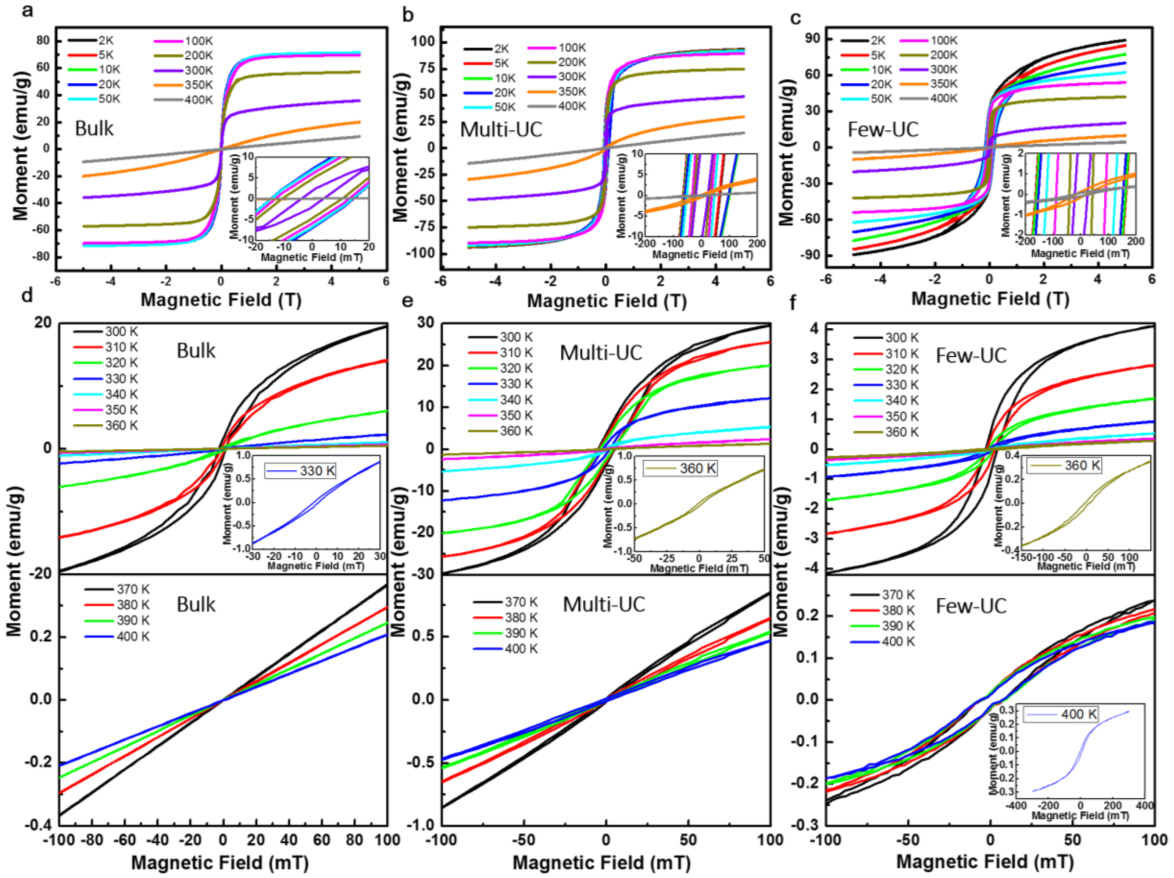

Supplementary Fig. 8. Magnetic properties of bulk, multi-UC and few-UC CrTe at different temperatures. a-c, M-H hysteresis loops of bulk (**a**), multi-UC (**b**) and few-UC (**c**) CrTe crystals from 2 to 400 K at a magnetic field range of -5~5 T. **d-e,** High temperature M-H hysteresis loops of bulk (**d**), multi-UC (**e**) and few-UC (**f**) CrTe crystals from 300 to 400 K at a magnetic field range of -0.3~0.3 T.

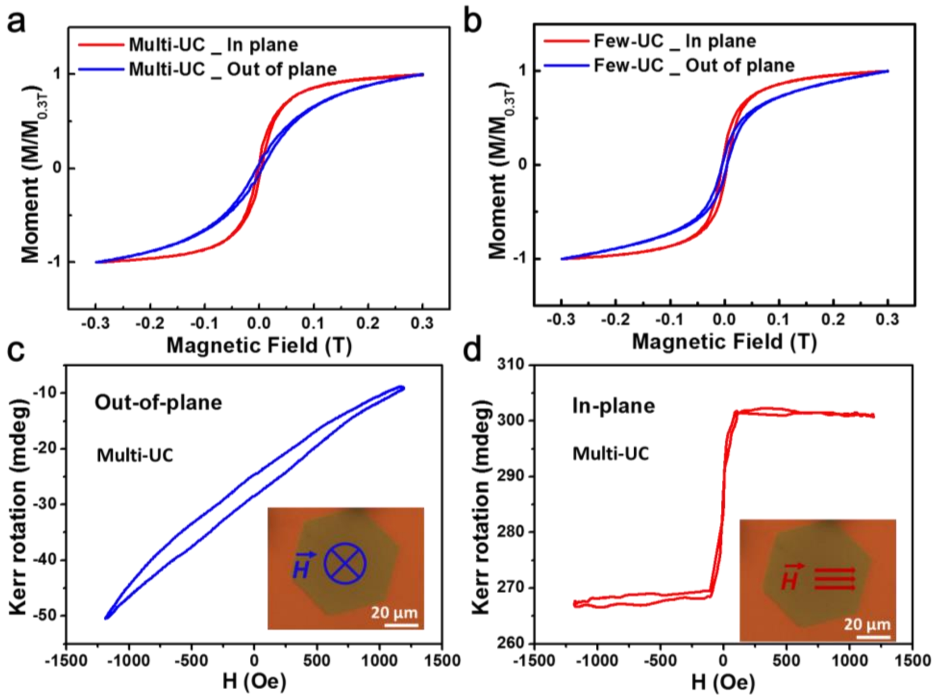

Supplementary Fig. 9. Magnetic anisotropy of multi- and few-UC CrTe crystals. a, b, The in-plane and out-of-plane M-H hysteresis loops for multi-UC (**a**) and few-UC (**b**) CrTe crystals at 300 K. **c, d,** The out-of-plane (**c**) and in-plane (**d**) Kerr rotation loops for a multi-UC CrTe crystal at 300 K.

Notes: Both multi-UC and few-UC CrTe crystals exhibit in-plane easy magnetization axis feature.

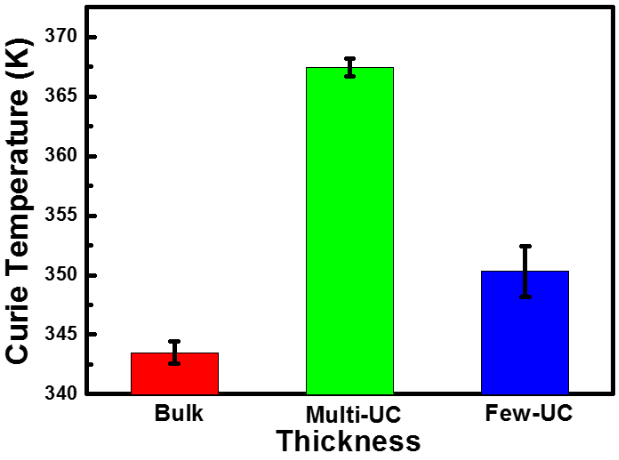

Supplementary Fig. 10. Curie temperature Tc of intrinsic ferromagnetism derived from VSM magnetization results for CrTe crystals with different thickness. Error bars sem; N=3.

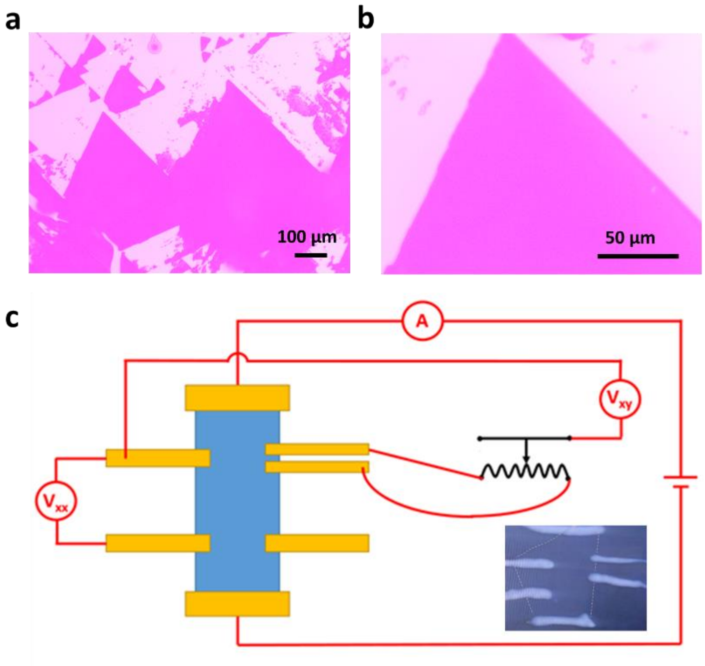

Supplementary Fig. 11. a, b, Optical photographs of the as-grown single crystals with a size of several hundred microns; **c,** Schematic diagram of five-wire Hall device. The inset is a photo of the Hall device made by dipping 25 µm gold wire in silver glue and pressing onto the sample.

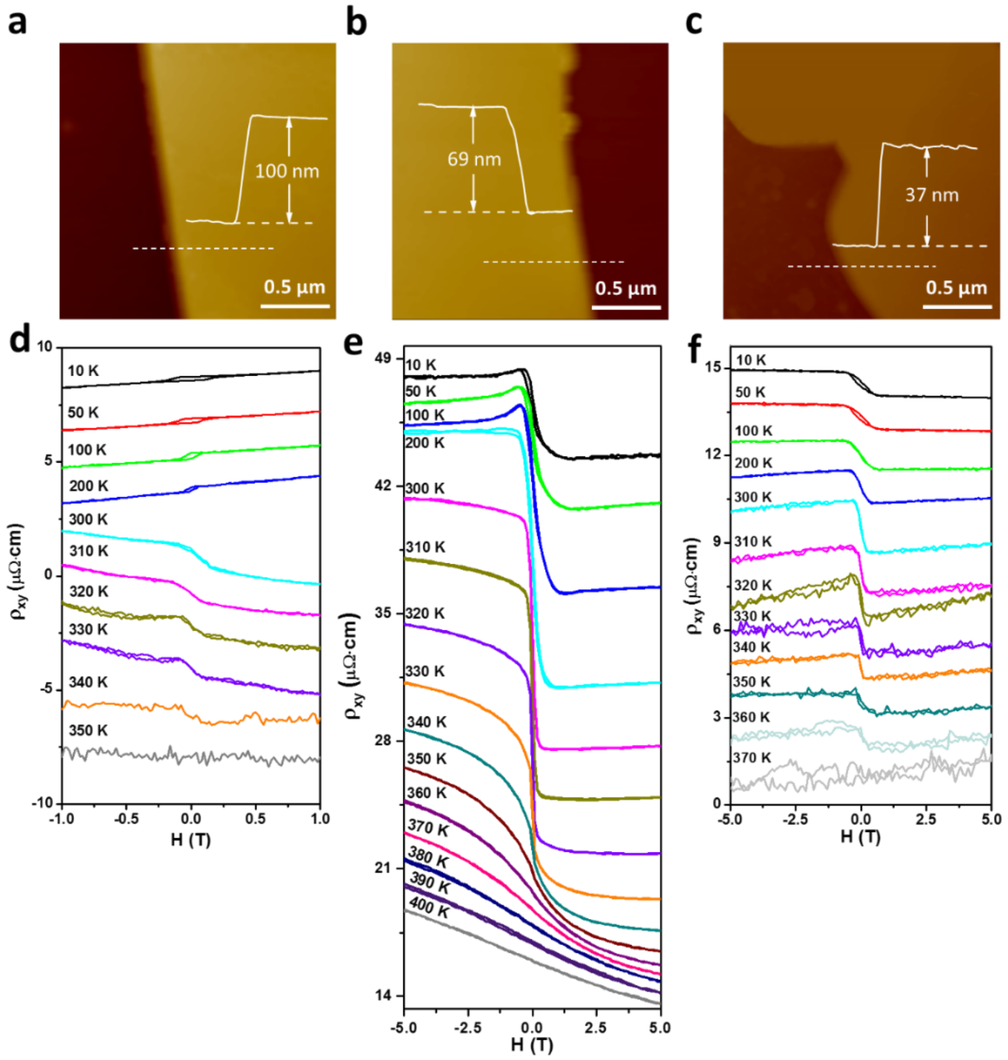

Supplementary Fig. 12. Anomalous Hall effect of single-sheet CrTe crystals with different thickness. a-c, AFM morphologies of CrTe crystals with three different thicknesses, the inset shows the corresponding thickness profiles; **d-f,** Anomalous Hall effect of 100 nm (**d**), 69 nm (**e**) and 37 nm (**f**) CrTe crystals in (**a-c**).

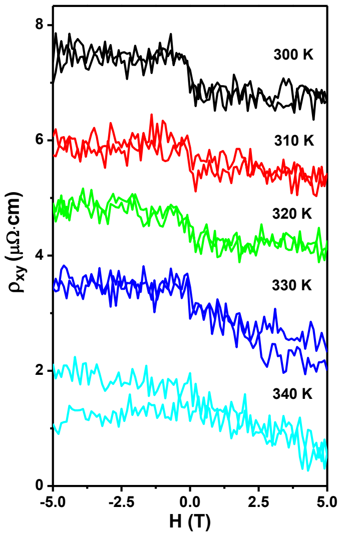

Supplementary Fig. 13. Anomalous Hall effect of bulk CrTe crystal. The thickness was determined to be ~20 μm by a micrometer.

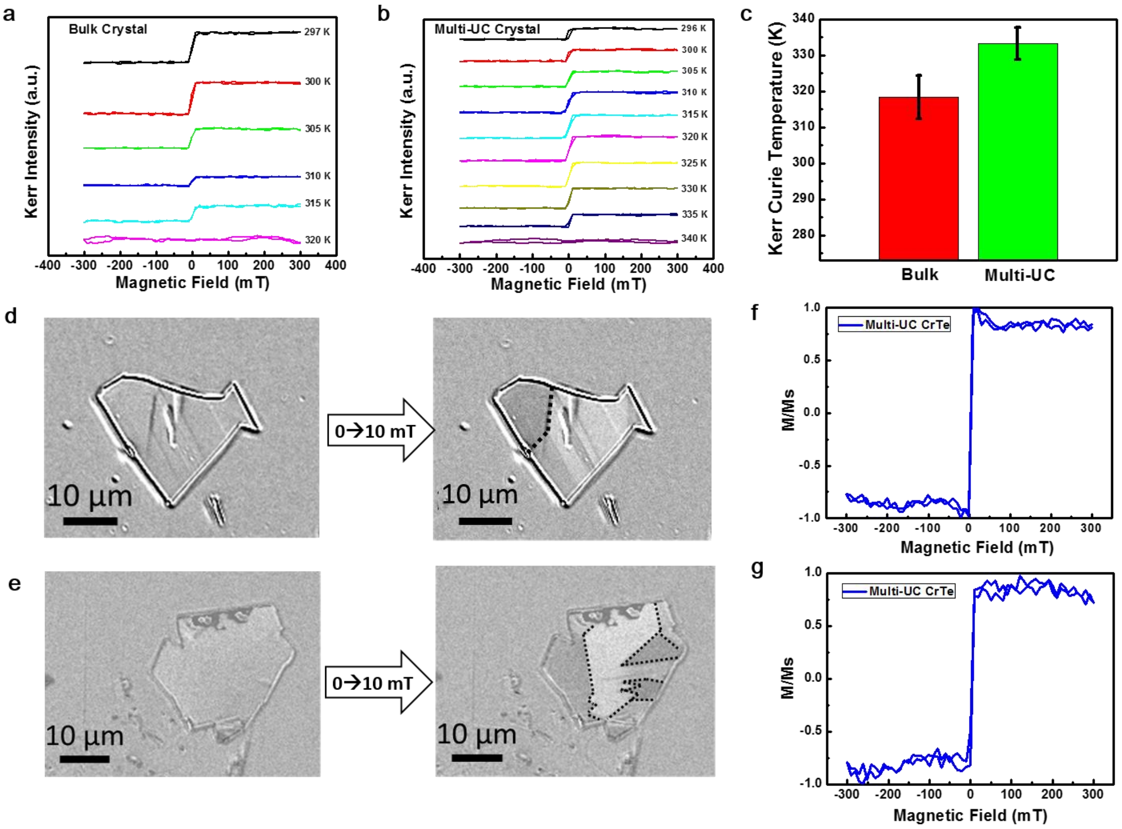

Supplementary Fig. 14. Single sheet magneto-optical Kerr effect tests and microscopy of single sheet bulk and multi-UC CrTe crystals, showing higher Tc in multi-UC than bulk CrTe crystals (a-c) and room temperature LDW magnetic domain wall behavior in multi-UC CrTe 2D crystal. a, b, Kerr intensity curves for a single sheet bulk (**a**, 1.1 µm, ~1375 UC) and multi-UC (**b**, 57 nm, ~71 UC) CrTe crystals at different temperatures measured by Evico magnetics Kerr Microscope & Magnetometer. **c,** Single sheet magnetoptical Kerr effect tests-derived Tc of bulk and multi-UC CrTe crystals by single sheet tests. Error bars sem; N=3. **d-e,** Room temperature magnetoptical Kerr images for two multi-UC CrTe single sheets at magnetic field from 0 mT to 10 mT, the dash lines shows the linear domain walls. Thickness of sheets in (**d**) and (**e**) is 57 nm (~71 UC) and 36 nm (~45 UC), respectively. **f, g,** Room temperature Kerr intensity curves collected from multi-UC CrTe single sheets in (**d**) and (**e**).

Notes:

(1) The difference of absolute Tc value from magnetoptical Kerr effect tests and that from VSM are mainly from sensitivity difference and testing modes of two methods. However, we see a similar trend with much higher Tc in multi-UC CrTe 2D crystals than that in bulk CrTe crystals from these single sheet magnetoptical Kerr effect tests, as shown in the VSM tests

indicating their reliability.

(2) Unfortunately, we find that single sheet magneto-optical Kerr effect tests of few-UC CrTe is beyond the sensitivity of our instrument for magneto-optical Kerr microscopy. Thus, we test single sheet few-UC crystals (~tri-UC to 10 UC) by MFM at room temperature and room temperature 2D ferromagnetism in few-UC has been confirmed by MFM tests (Fig. 4). However, single sheet mono-UC MFM tests fail due to the weak MFM signals for mono-UC crystals. As first principles-calculations (Supplementary Fig. 20) indicate that, when compared with bulk CrTe crystals, significantly enhanced spin plolarization and ferromagnetism in mono-UC CrTe is more like that in ferromagnetic few-UC CrTe, quite different from bulk CrTe crystals. Therefore room temperature ferromagnetism like few-UC CrTe should be highly expected in mono-UC CrTe.

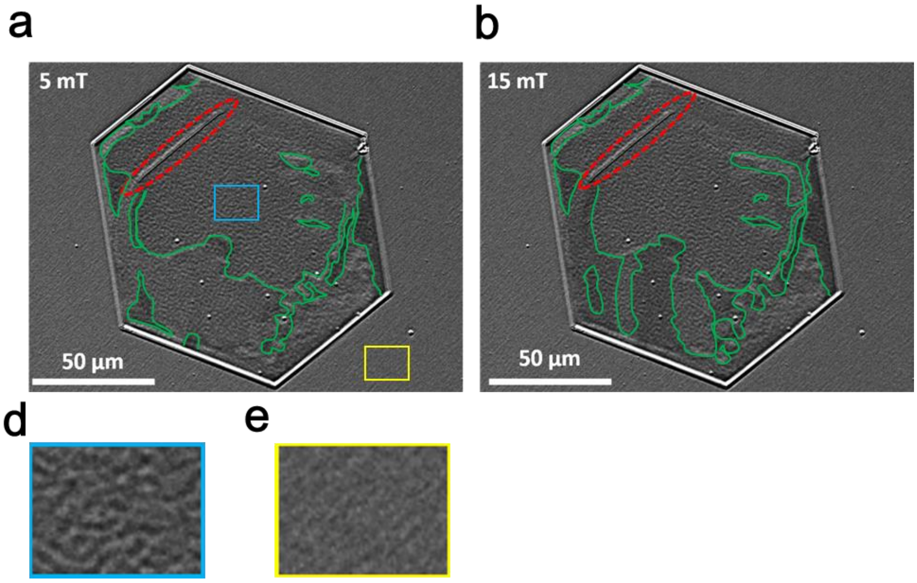

Supplementary Fig. 15. Room temperature magneto-optical tests of a single sheet bulk CrTe crystal. a, b, Kerr intensity images of a bulk CrTe crystal under magnetic fields of 5 mT (a) and 15 mT (**b**), showing random domain walls and no LDW found. Thickness of bulk CrTe crystal is ~5.1 µm (~6375 UC). Note that the bight line in the red dotted circle is the high reflection line caused by the morphology of the sample, not the domain wall. The green solid lines represent random magnetic domain walls, that separate different domains. **d, e,** The fine domain structure on sample (**d**, blue box in **a**) and substrate (**e**, yellow box in **a**).

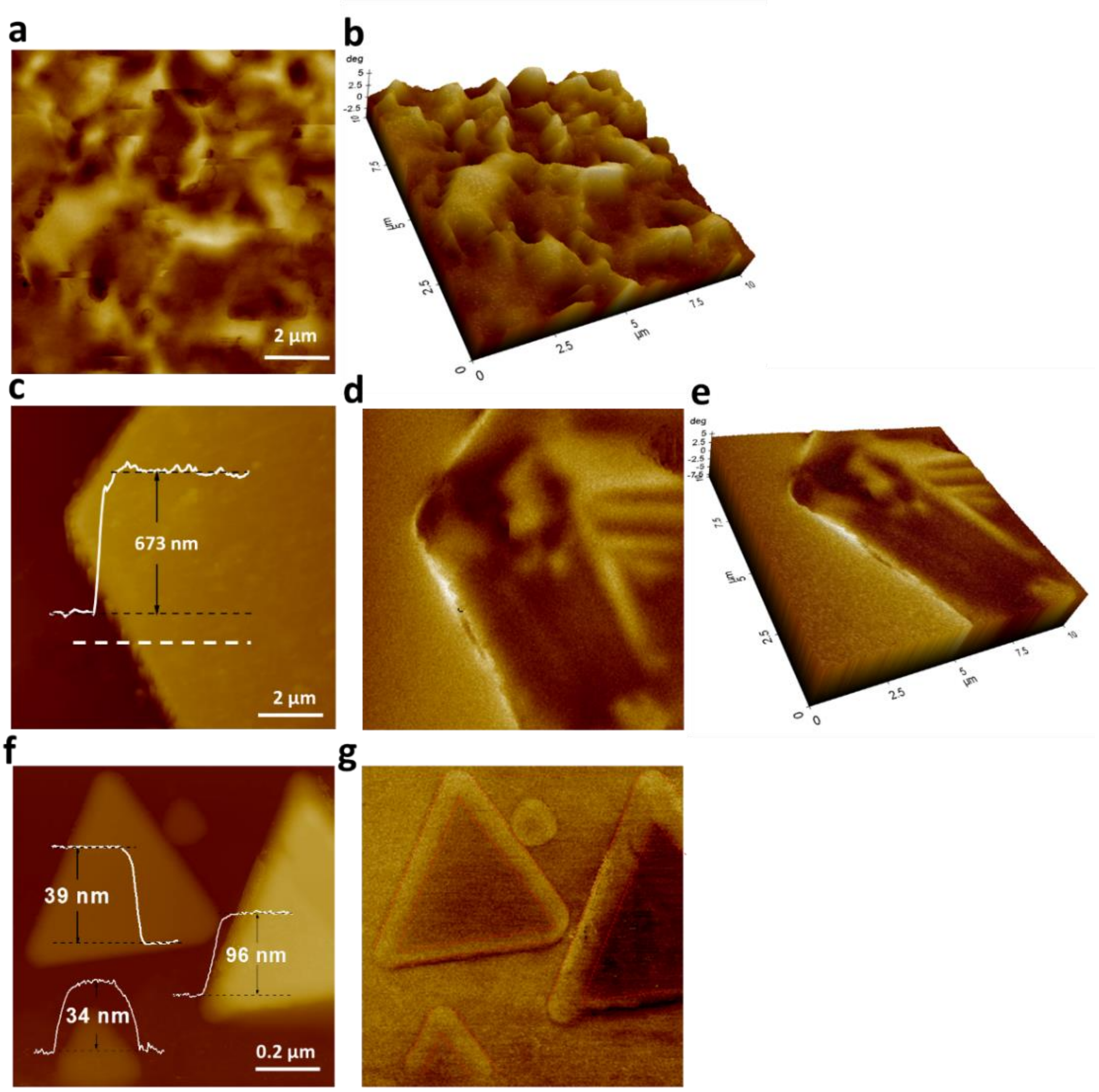

Supplementary Fig. 16. Room temperature MFM magnetic domain imaging of CrTe single crystals with different thicknesses. a, b, MFM phase image (**a**) and corresponding 3D view (**b**) of a 20 µm thick CrTe crystal; **c-e,** AFM image (**c**), MFM phase image (**d**) and corresponding 3D view (**e**) of a 673 nm thick CrTe crystal; **f, g**, AFM image (**f**) and MFM phase image (**g**) of CrTe crystals with thickness of 34, 39 and 96 nm.

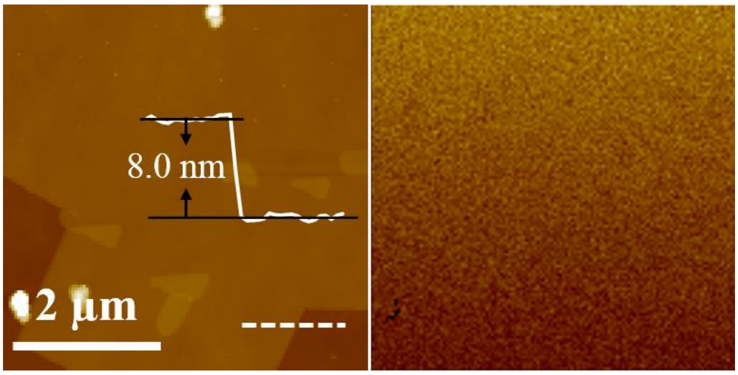

Supplementary Fig. 17. AFM Topography image (left) of an 8 nm thick few-UC CrTe crystal (~ 10 UC) with corresponding in site MFM phase image (right) with non-pre-magnetized AFM tip. The AFM Tip is not pre-magnetized and thus there is no identifiable MFM phase signal of the 8 nm thick CrTe crystal. No external magnetic field is applied on few-UC CrTe during the MFM tests.

Notes:

To confirm MFM phase signals from the ferromagnetic property of CrTe, a non-pre-magnetized AFM tip is also applied for a ~8 nm thick (~ 10 UC) few-UC CrTe at the same set distance and no identifiable MFM phase signal is found (Supplementary Fig. 17), indicating that the set distance is large enough to preclude short-range atomic forces and the MFM phase signal in Fig. 4a is induced by magnetic force between the sample and pre-magnetized tip.

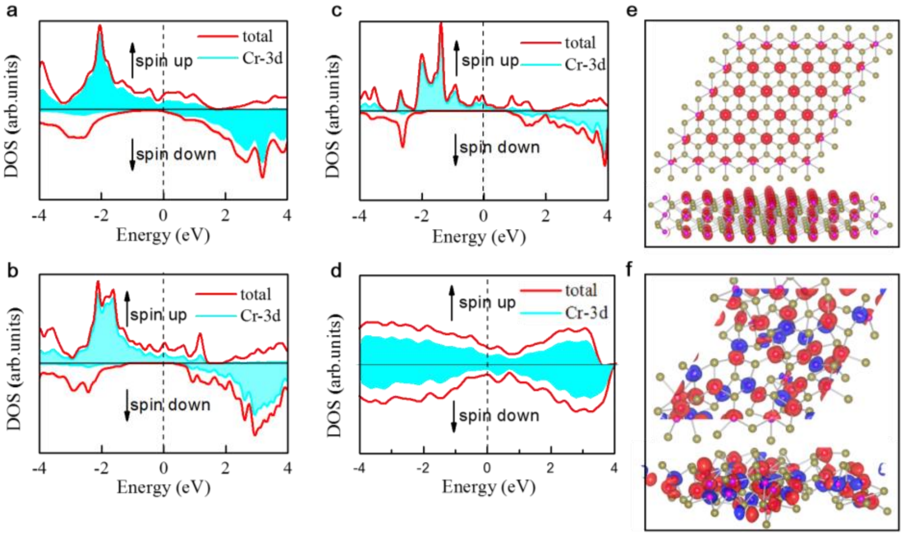

Supplementary Fig. 18. The spin-resolved density of states (DOSs) and spin density distributions of bulk, multi-UC, few-UC and disordered few-UC CrTe crystals. **a-d,** Total DOS and partial DOS of Cr-3d states in bulk (**a**), multi-UC (**b**), few-UC (**c**) and disordered few-UC (**d**) CrTe crystals. The vertical dash lines denote the position of Fermi level. **e, f,** Spin density distribution of ordered few-UC (**e**) and disordered few-UC (**f**) CrTe crystal. The red and blue isosurfaces represent positive and negative spin density, respectively.

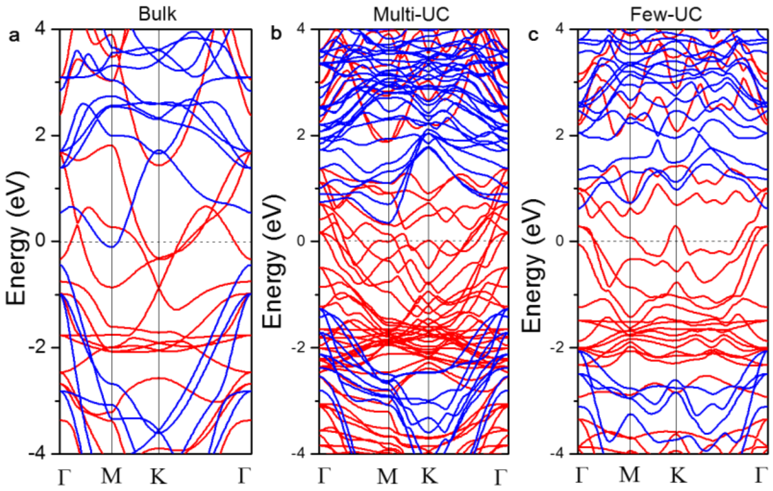

Supplementary Fig. 19. Spin-polarized band structures of bulk (a), multi-UC (b) and few-UC (c) CrTe crystals. The red dot lines and blue solid lines represent the majority-spin and minority-spin bands, respectively. The horizontal dash lines denote the position of Fermi level.

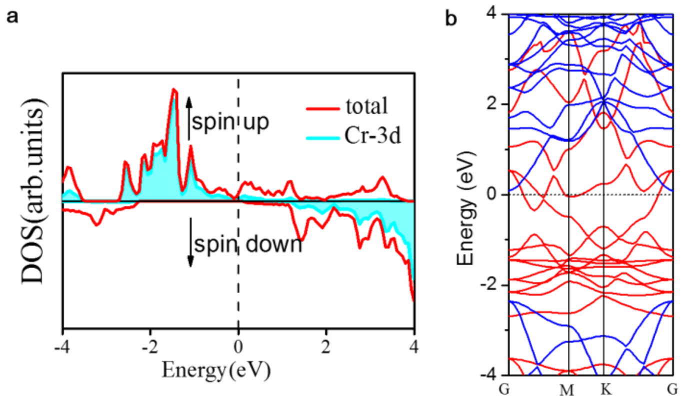

Supplementary Fig. 20. The spin-resolved density of states (DOSs) (a) and band structures (b) of mono-UC CrTe crystals.

Notes: First principles-calculations indicate that, when compared with bulk CrTe crystals, enhanced spin plolarization and ferromagnetism in mono-UC CrTe is more like that in ferromagnetic few-UC CrTe. Therefore, intrinsic room temperature 2D ferromagnetism is highly expected in mono-UC CrTe, although direct tests are difficult for mono-UC because of the difficulties to get large scale preparation for VSM test or have enough single sheet microscopy instrument sensitivity for direct single sheet test, like many monolayer cases in van der Waals 2D crystals[1].

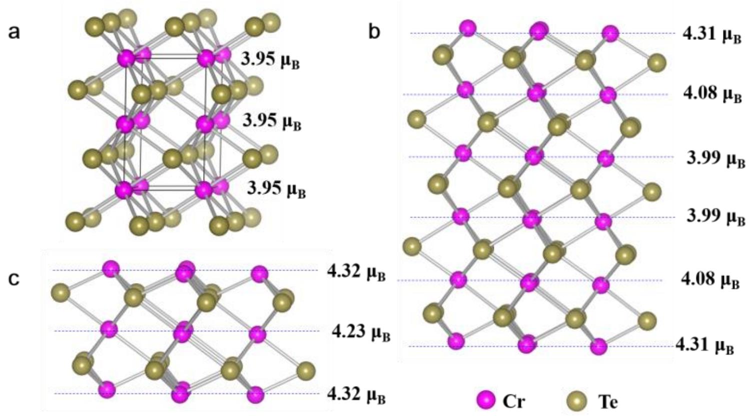

Supplementary Fig. 21. The calculated atomic magnetic moments of bulk (a), multi-UC (b) and few-UC (c) CrTe crystals.

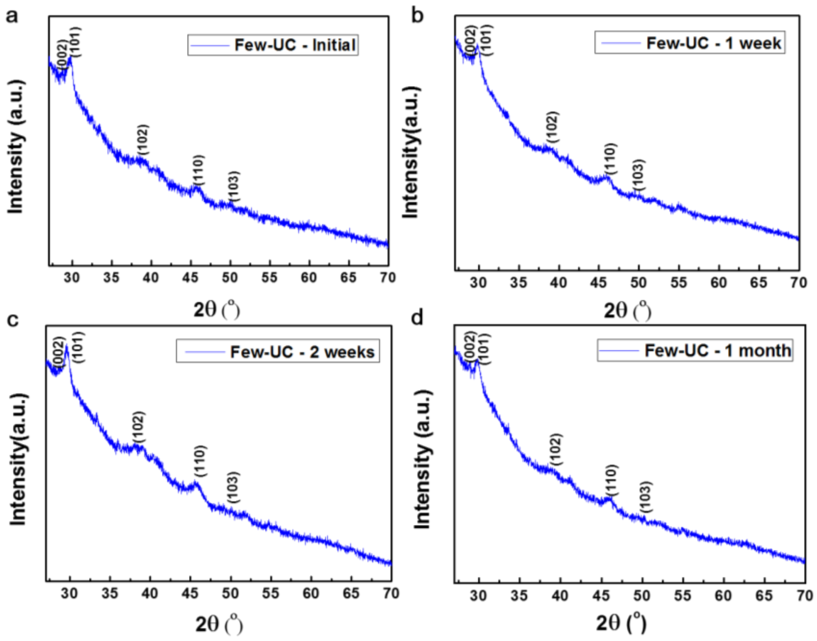

Supplementary Fig. 22. Crystal stability of few-UC CrTe crystals in ambient air. a-d, XRD spectrum of few-UC CrTe crystals put in the ambient air for 0 (**a**), 7 (**b**), 14 (**c**) and 30 (**d**) days.

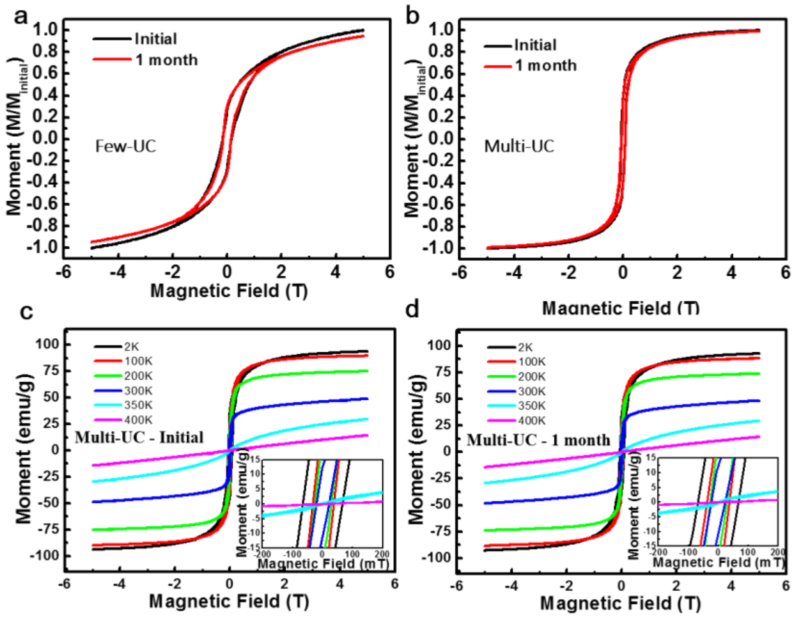

Supplementary Fig. 23. Stability of ferromagnetism in few- and multi-UC CrTe crystals under long-term ambient air exposure. a, b, The M-H hysteresis measurement of few-UC (**a**) and multi-UC (**b**) CrTe crystals for pristine crystal and crystals with 1 month exposure in ambient air condition at 2 K. **c, d,** M-H hysteresis measurement of multi-UC CrTe crystals from 2 to 400 K for pristine crystal and crystals with one month exposure in ambient air condition.

Supplementary Table 1 | Saturation magnetic moment and coercivity of bulk, multi-UC, few-UC CrTe crystals and traditional ferromagnetic materials Fe, Co, Ni, and $BaFe_{12}O_{19}$ as used in Fig. 3g-2i.

Materials	Samples	Moment (emu/g)		Moment (μB)		Hc (Oe)	
		2 K	300 K	2 K	300 K	2 K	300 K
Fe thin foil	S1	234.00	226.00	2.3388	2.2588	105.5	99.4
	S2	233.85	227.40	2.3373	2.2728	199.9	197.5
	S3	239.07	235.16	2.3894	2.3504	86.9	86.3
Co thin foil	S1	166.80	167.60	1.7597	1.7597	426.1	247.8
	S2	161.47	160.24	1.7035	1.7035	219.6	152.6
	S3	162.18	161.12	1.7110	1.7110	218.5	153.8
Ni thin foil	S1	55.74	52.45	0.5861	0.5515	118.3	105.8
	S2	59.99	56.19	0.6307	0.5908	113.5	105.5
	S3	58.72	54.73	0.6174	0.5754	149.1	133.8
$BaFe_{12}O_{19}$ nanoparticles	S1	96.11	68.37	1.5937	1.1337	803.0	750.0
	S2	96.42	68.08	1.5988	1.1289	780.0	765.0
	S3	105.98	74.64	1.7573	1.2376	786.0	735.0
Bulk CrTe	S1	70.47	35.96	2.2670	1.1568	147.00	37.00
	S2	68.27	39.83	2.1962	1.2813	331.81	175.35
	S3	69.94	40.82	2.2499	1.3132	342.22	171.64
Multi-UC CrTe	S1	93.71	48.89	3.0146	1.5728	655.64	215.00
	S2	79.67	44.87	2.5630	1.4434	653.52	337.93
	S3	63.70	39.91	2.0492	1.2839	660.77	231.54
Few-UC CrTe	S1	89.43	20.29	2.8769	0.6527	1629.5	229.0
	S2	42.82	22.68	1.3775	0.7296	1632.9	220.7
	S3	47.78	10.91	1.5371	0.3510	2015.5	213.6

Notes:

Commercial Fe, Co and Ni thin foils (size ~2 x 1 x 0.2 mm) are used for direct test here. For nanoscale Fe, Co, Ni, the magnetic moments of Fe, Co, Ni nanoparticles were reported to be ~3.0, 2.3 and 1.0 μB for clusters of less than 50 atoms at low temperature[2]. The magnetic moments of multi- and few-UC CrTe crystals are still comparable with that of these nanoscale Fe, Co, Ni. $BaFe_{12}O_{19}$ tested is widely commercial available nanoparticles with diameter ~200 nm.

As for the coercivity, the reported ultrathin Fe, Co, Ni thin films show different values

depending on substrates and some typical values are listed below: less than 24 Oe for 5.5 nm Fe thin film at room temperature[3]; 280-350 Oe for 1.52 nm Co thin film at 10 K[4], less than 0.5 Oe for 2.6 nm Ni film at 77 K[5] and 130-250 Oe at 300 K for 14-66 nm Ni film[6]. The coercivity of few-UC 2D CrTe crystals is about over 9 times that of ultrathin Fe at room temperature but comparable coercivity with Co and Ni ultrathin films at low temperature or 300 K.

Such strong intrinsic room temperature ferromagnetism comparable with widely used Fe, Co, Ni, and $BaFe_{12}O_{19}$ imply a great potential of freestanding ultrathin 2D CrTe crystals in practical applications. The tunable coercivity by thickness and dimension in freestanding 2D CrTe crystals is also very important for its practical applications when combining with its freestanding 2D crystal states and strong intrinsic room temperature and beyond room temperature 2D ferromagnetism.

Supplementary Discussions:

1. Comparison of freestanding 2D CrTe crystals with non-freestanding traditional CrTe ultrathin film on substrate

Freestanding 2D CrTe crystals show room temperature 2D ferromagnetism with Tc up to 367 K, while traditional CrTe ultrathin films on $SrTiO_3$ substrate show a low Curie temperature much lower than room temperature (Tc= 200 K for 6 nm thick CrTe films)[7]. The difference may come from the freestanding states and materials parameters such as lattice constant (a=4.03 Å for freestanding 2D CrTe crystals and a=3.95 Å for traditional CrTe ultrathin film on substrates) and crystal growth direction. These results indicate freestanding non-van der Waals 2D crystals provide a new route to obtain and tune the intrinsic room-temperature 2D ferromagnetism in 2D crystals, making it easier and more possible to achieve very different physical properties from traditional ultrathin films on substrates.

2. Spontaneous magnetization (B=0) and spontaneous spin polarization in freestanding 2D CrTe crystals

As shown in main text, spontaneous magnetization (B=0) and spontaneous spin polarization in few- and multi-UC CrTe crystals is ~4.7 and 2.3 times that of bulk CrTe crystal, respectively (Fig. 3d), considering that the spontaneous magnetization (B=0) is highly related with the spontaneous spin polarization of the carriers or conduction electrons[8,9]. The results imply a high spontaneous spin polarization in few- and multi-UC CrTe crystals than that in bulk CrTe crystals, which is consistent with calculations. The calculated spin-resolved density of states (DOSs) and band structures indicate that the spin polarization in few- and multi-UC CrTe

crystals are significantly enhanced, especially for electronic states around Fermi level (see Supplementary Figs. 18-20). Therefore the results here may provide a new route to tune and achieve high spin polarization in 2D ferromagnetic crystals and may make new quantum devices possible based on spontaneous spin polarization in 2D crystals.

Supplementary References

1. Gong, C. *et al.* Discovery of intrinsic ferromagnetism in two-dimensional van der Waals crystals. *Nature* **546,** 265-269 (2017).
2. Billas, I. M. L., Châtelain, A. & Heer, W. A. d. Magnetism from the Atom to the Bulk in Iron, Cobalt, and Nickel Clusters. *Science* **265,** 1682-1684 (1994).
3. Lee, W. Y., Choi, B.-C., Xu, Y. B. & Bland, J. A. C. Magnetization reversal dynamics in epitaxial Fe_GaAs(001) thin films. *Phys. Rev. B* **60,** 10216-10221 (1999).
4. Chappert, C. & Bruno, P. Magnetic anisotropy in metallic ultrathin films and related experiments on cobalt films. *J. Appl. Phys.* **64,** 5736-5741 (1988).
5. Snigirev, O. V., Andreev, K. E. & Tishin, A. M. Magnetic properties of thin Ni films measured by a dc SQUID-based magnetic microscope. *Phys. Rev. B* **55,** 14429-14433 (1997).
6. Ausanio, G. *et al.* Magnetic and morphological characteristics of nickel nanoparticles films produced by femtosecond laser ablation. *Appl. Phys. Lett.* **85,** 4103-4105 (2004).
7. Zhao, D. *et al.* Observation of unconventional anomalous Hall effect in epitaxial CrTe thin films. *Nano Res.* **11,** 3116-3121 (2018).
8. Smit, J. The spontaneous hall effect in ferromagnetics II. *Physica* **24,** 39-51 (1958).
9. Thiel, L. *et al.* Probing magnetism in 2D materials at the nanoscale with single-spin microscopy. *Science* **364,** 973-976 (2019).
